# Importance of Mediator complex in the regulation and integration of diverse signaling pathways in plants

**DOI:** 10.3389/fpls.2015.00757

**Published:** 2015-09-17

**Authors:** Subhasis Samanta, Jitendra K. Thakur

**Affiliations:** Plant Mediator Lab, National Institute of Plant Genome ResearchNew Delhi, India

**Keywords:** transcription, RNA polymerase II, mediator complex, development, defense signaling, abiotic stress, Arabidopsis, rice

## Abstract

Basic transcriptional machinery in eukaryotes is assisted by a number of cofactors, which either increase or decrease the rate of transcription. Mediator complex is one such cofactor, and recently has drawn a lot of interest because of its integrative power to converge different signaling pathways before channeling the transcription instructions to the RNA polymerase II machinery. Like yeast and metazoans, plants do possess the Mediator complex across the kingdom, and its isolation and subunit analyses have been reported from the model plant, Arabidopsis. Genetic, and molecular analyses have unraveled important regulatory roles of Mediator subunits at every stage of plant life cycle starting from flowering to embryo and organ development, to even size determination. It also contributes immensely to the survival of plants against different environmental vagaries by the timely activation of its resistance mechanisms. Here, we have provided an overview of plant Mediator complex starting from its discovery to regulation of stoichiometry of its subunits. We have also reviewed involvement of different Mediator subunits in different processes and pathways including defense response pathways evoked by diverse biotic cues. Wherever possible, attempts have been made to provide mechanistic insight of Mediator's involvement in these processes.

## Introduction

**Figure 1:**
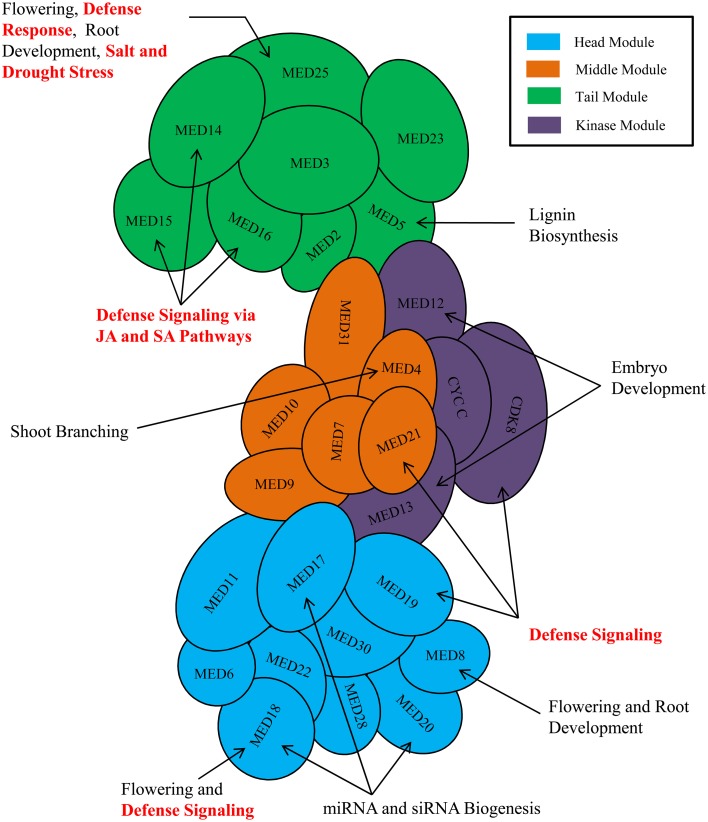
**Involvement of plant Mediator subunits in development, and various biotic and abiotic stress responses**. Arrangement of Mediator subunits is modular in nature. Mediator complex subunits are arranged into four modules-Head module (Cyan), Middle module (Orange), Tail module (Green), and a separable Kinase module (Purple). Only the known and important functions of plant Mediator complex are shown in the figure. MED26 and plant-specific Mediator subunits (MED34, MED35, MED36, and MED37) are not shown in the figure because of insufficient information about their module positions.

The process of transcription in eukaryotic organism is an immensely complex and highly orchestrated phenomenon, and is mediated by a plethora of proteins wherein primary role is played by RNA polymerase II (RNAP II) (Lee and Young, 2000). The process is regulated both at the transcription initiation and elongation stages by a seemingly endless collections of regulatory proteins involved in different mechanisms (Woychik and Hampsey, 2002). Over the past 30 years, elegant biochemical, genetic, and structural biology works have established a core set of six general transcription factors (TFIIA, TFIIB, TFIID, TFIIE, TFIIF, and TFIIH) along with RNAP II as the core elements, which are obligatory to initiate and sustain any successful gene transcription event. On the other hand, among the numerous co-activators characterized till date to facilitate the initial recruitment of RNAP II to the core promoter and the subsequent transcript elongation, the Mediator complex has emerged as potentially the most crucial by virtue of its essentiality in RNAP II-mediated transcription (Myers and Kornberg, 2000; Conaway et al., 2005; Kornberg, 2005; Malik and Roeder, 2010). The Mediator complex is a highly conserved and integral part of RNAP II-mediated transcriptional machinery of the eukaryotes. In the past, the composition of the Mediator complex and the functions of different Mediator subunits have been reviewed several times focusing on yeasts and metazoans. In plant biology, the central role of Mediator complex in RNAP II-mediated transcriptional event has already been recognized by its discovery in Arabidopsis and other crop plants. The recent time has experienced a flush of interesting reports on the plant Mediator subunits detailing its quintessential role not only in growth and developmental processes, but also in biotic and abiotic stress responses (Figure 1). Realizing the need for an updated and critical analysis of roles of Mediator subunits in plants' life, this review summarizes the functions of Mediator subunits and provides insight about the depth and complexity of involvement of Mediator complex in transcriptional regulations of plant genes.

## Discovery of mediator subunits in plants

Mediator complex was first discovered in yeast in 1990, and within few years also reported in human (Kelleher et al., 1990; Flanagan et al., 1991; Thompson et al., 1993; Kim et al., 1994; Fondell et al., 1996; Ito et al., 1999). It took more than a decade to purify and characterize first Mediator complex from Arabidopsis cell suspension culture (Bäckström et al., 2007). Bioinformatics predictions including 16 plant species representing the entire plant kingdom ascertained its existence in major crop species including rice (Mathur et al., 2011). Despite low sequence similarity among the orthologs of the Mediator subunits in different organisms because of its rapid evolution, orthologs of all the yeast Mediator subunits reported to be present in plants too (Levine and Tjian, 2003; Bourbon, 2008; Mathur et al., 2011). MED1 is not found in higher plants, but is encoded by the genome of red algae. Also, many of the subunits which were earlier reported to be plant-specific, are actually present in organisms of other kingdom (Bäckström et al., 2007; Bourbon, 2008; Mathur et al., 2011). Thus, most of the Mediator subunits are conserved across the eukaryotic organisms. Structure of Mediator complexes from different organisms have been analyzed with the help of electron microscopy and seemed to be astonishingly similar (Cai et al., 2009; Tsai et al., 2014; Wang et al., 2014). Alignment of secondary structures of the individual plant Mediator subunits with orthologs in other organisms also suggests quite high structural resemblance (Mathur et al., 2011). However, as in the case of many other proteins, plant genomes code for more number of paralogs of several Mediator subunits. Four paralogs of MED15 are encoded by the genes present in *MED15* cluster on chromosome 1 of Arabidopsis (Pasrija and Thakur, 2012). Though functional significance of the presence of multiple paralogs of a particular Mediator subunit is not demonstrated yet, they might help in broadening the regulatory capability of the complex. The spatio-temporal regulation of the expression level of different paralogs of a particular Mediator subunit can make the Mediator structure more dynamic depending upon the external milieu and the growth and developmental phase of the plant.

## Modular organization of mediator complex and its functions

Mediator is a multi-protein conglomerate, which is enormous in size and complex in composition. The individual protein identity is termed as MED subunit, and the numbers can vary from 25 to 30 depending upon the species. Another salient feature of the Mediator complex is its modular structure. The entire array of subunits of the Mediator complex is arranged into three modular structures; head module, middle module, and tail module (Asturias et al., 1999; Dotson et al., 2000; Chadick and Asturias, 2005; Bourbon, 2008). These three modules together form the Mediator core. The RNAP II-bound Mediator complex is called “Holoenzyme.” In addition, there is also a separable kinase or CDK8 module in the Mediator complex, which consists of CDK8, cyclin C, MED12, and MED13 (Wang et al., 2001; Spahr et al., 2003; Elmlund et al., 2006). The Mediator core associates with RNAP II favoring transcription whereas the kinase module-bound Mediator complex dissociates from RNAP II to repress transcription. The Mediator can shuttle between these two different forms (Mediator core and Mediator core-kinase complex) depending upon the cellular contexts. It is worth mentioning here that the kinase or the CDK module subunits were characteristically absent from the first-ever Mediator complex purified from Arabidopsis (Bäckström et al., 2007). The mechanism of Mediator functioning is manifested in different ways. Mediator acts as a bridge between the *cis*-element bound transcription factors and the promoter bound RNAP II, hence recruits the RNAP II machinery to the promoter of the transcriptionally active genes. However, recent progress suggests that Mediator is not just an adaptor molecule between the transcription factor and the basic transcriptional machinery, but provides a platform for recruitment of other cofactors, GTFs, and TFs for the formation of Pre-Initiation Complex (PIC). These interactions can bring changes in the structure of the resultant complex which may affect transcription. Thus, Mediator acts as a docking site for many other transcriptional regulators and plays critical role in relaying regulatory signals from them to RNAP II machinery (Takahashi et al., 2011, 2015).

In the beginning, Mediator complex was thought to be involved only in the initiation step of transcription as evident by its interaction with the components of the transcription initiation complex (Mittler et al., 2001; Baek et al., 2002; Cantin et al., 2003; Johnson and Carey, 2003; Wang et al., 2005). However, in last few years, Mediator has been reported to be involved in the regulation of many other steps of transcription like promoter escape (Malik et al., 2007; Cheng et al., 2012; Jishage et al., 2012), elongation (Takahashi et al., 2011; Conaway and Conaway, 2013; Galbraith et al., 2013), termination (Mukundan and Ansari, 2011, 2013), as well as in other co-transcriptional RNA processing events (Kim et al., 2011; Huang et al., 2012; Oya et al., 2013). Mediator has also been implicated in epigenetic and architectural modification of chromatin leading to changes in gene expression (Kagey et al., 2010; Zhu et al., 2011; Fukasawa et al., 2012; Liu and Myers, 2012; Lai et al., 2013; Tsutsui et al., 2013; Zhang et al., 2013a). Thus, it seems that Mediator is critical for almost every aspect of transcription of eukaryotic genes.

Mediator complex was first discovered as an entity required for enhanced transcription of an *in vitro* transcription system which contained RNAP II and essential general transcription factors (Flanagan et al., 1991; Kim et al., 1994). Now, it is well-established that a number of transcription factors need Mediator to enhance the process of transcription. At this point of time, it is not clear if this positive effect in the activation of transcription is direct or indirect. However, the role of Mediator complex in the repression of gene expression has also been reported in many cases. The repressor activity of Mediator is primarily attributed to the kinase module. Association of this module with the core Mediator complex occludes the RNAP II from the PIC exerting a repressive role in the RNAP II-mediated transcriptional events (Holstege et al., 1998; Samuelsen et al., 2003; Elmlund et al., 2006; Knuesel et al., 2009). However, contrary to this, researchers from different laboratories have also reported the positive effect of kinase module on gene expression (Donner et al., 2007, 2010; Belakavadi and Fondell, 2010). Mechanistically, the activation property of CDK8 module may partly be attributed to its ability to recruit transcription elongation factor, P-TEFb, and to release the promoter-proximally paused RNAP II into productive elongation phase (Takahashi et al., 2011, 2015). The added complexity of the Mediator functions is brought about by the presence of multiple isoforms of the kinase module, which might help the Mediator complex to fine-tune the gene expression in a tissue, cell, or even pathway specific manner (Sato et al., 2004; Bourbon, 2008; Conaway and Conaway, 2011; Mathur et al., 2011).

## Mediator as a global regulator of gene expression vs. its gene selective functions

Even after two decades of its discovery, it is still debatable whether Mediator complex is a general transcription factor or just a cofactor of gene expression. Although, initially identified as an entity that supports activator-dependent transcription, now, according to several evidences, Mediator complex can also be categorized as general transcription factor. The human Mediator complex can support basal level transcription of many genes by playing important roles in assembly of PIC and transcription initiation (Mittler et al., 2001; Baek et al., 2002). Mediator complex enhances the RNAP II recruitment to the protein coding genes and provides stability to the transcription machinery assembled at the promoter region (Cantin et al., 2003; Baek et al., 2006). In yeast, deletion of *MED17* makes Mediator structurally unstable, and in the conditional yeast *med17* knockout expression of protein-coding genes is severely compromised on a genome-wide scale (Thompson and Young, 1995; Ansari et al., 2009). In plants, comparison of transcriptomes between *nrpb2-3* (mutant in second largest subunit of RNAP II) and *med20a* plants revealed 84% overlap in the down-regulated genes, implying that Mediator complex is as important as the RNAP II for gene expression (Kim et al., 2011). Thus, the Mediator complex should be regarded as an integral component of the basal transcriptional machinery in the eukaryotes, and the roles are manifested in the forms of RNAP II recruitment and activation, co-ordination of PIC assembly, control of TFIIH-dependent RNAP II CTD phosphorylation within the PIC, and sustained or transient repression of transcription initiation via Mediator-CDK8 module interactions (Taatjes, 2010). Nevertheless, increasing number of reports of deletions of certain Mediator subunits affecting particular phenotype suggest that several individual Mediator subunits possess specific functions as well (Tables 1–**3**). This dilemma could be explained by taking into account the modular nature of Mediator complex and by assigning the “division of labor” principles to each module. The head module subunits might be involved in the more basic functions of the Mediator complex, whereas the tail module subunits residing on the periphery might be controlling gene-specific functions by contacting the specific transcription factors.

**Table 1:** **Plant Mediator subunits involved in growth and development**.

**Growth and development**	**Gene name**	**Functions in brief**	**Interacting proteins**	**References**
Embryonic development	*MED12/GCT*	Mediates embryo pattern formation repressing the transcriptional program mediated by KANADI 1 and KANADI 2	Unknown	Gillmor et al., 2010, 2014
	*MED13/CCT/Macchi Bou2 (MAB2)*	Mediates embryo pattern formation. Additionally, involved in auxin signaling	Unknown	Gillmor et al., 2010, 2014; Ito et al., 2011
Flower development	*AtMED25/PFT1*	Positively controls Constant (*CO*) and *FT* expression, promotes self-destruction by proteasome-mediated degradation	MBR1 and MBR2	Inigo et al., 2012a,b
	*AtMED8*	Involved in flowering. Transcript of *FLC* and *FT* were high and low, respectively in *Atmed8*	Unknown	Kidd et al., 2009
	*AtMED12*	Positive regulator of flowering	Unknown	Imura et al., 2012
	*AtMED18*	Has role in flowering. Binds with the promoter of *FLC* along with AtSUF4, keeps the expression level of *FLC* under control	AtSUF4	Zheng et al., 2013; Lai et al., 2014
Root development	*AtMED25*	The mutant is root hairless. The expression level of class III peroxidases got affected	Unknown	Sundaravelpandian et al., 2013
	*AtMED8*	Mechanistically similar to MED25, but may be in a different pathway	Unknown	Sundaravelpandian et al., 2013
Other growth and developmental events	*AtMED25/PFT1*	Mutants form large organs, partly because of increasing expression levels of expansions genes, *AtEXP1, AtEXP3, AtEXP5, AtEXP9, AtEXP11*, and *AtEXPB3*	Unknown	Xu and Li, 2011
	*AtMED8*	Positively controls the organ size	Unknown	Xu and Li, 2012
	*AtMED14/SWP*	The mutant has reduced leaf number and size, and disorganized SAM	LEUNIG; SMP1 and SMP2 (Probable)	Autran et al., 2002; Clay and Nelson, 2005; Gonzalez et al., 2007
	*AtMED18*	The mutant has floral deformities. It controls expression of floral homeotic genes like *AP1, PI*, and *AG*	Unknown	Kim et al., 2011; Zheng et al., 2013
	*AtMED16*	Regulates iron homeostasis in plants. Controls marker genes of iron homeostasis like *IRT1, FRO2*	AtMED25, FIT	Yang et al., 2014; Zhang et al., 2014
	*OsMED4*	Mutants are embryonic lethal. Speculated to be involved in rice tiller growth	OsSAD1 (RPA 34.5)	Li et al., 2014
	*AtCDK8/HEN3*	Controls the specification of stamen and carpel. Has an inhibitory role on *AG, AP1*, and *AP2* expression	CTD domain of RNAP II	Wang and Chen, 2004
	*AtMED5a* and *AtMED5b*	Involved in active transcriptional processes which inhibit growth and lignin biosynthesis in plants	Unknown	Bonawitz et al., 2012, 2014

## Functional analyses of mediator subunit genes

### Expression analyses of the mediator subunit genes

Tight transcriptional regulation of gene expression is very important for proper growth and development of plant and its protection from adverse environmental conditions. After the basic transcriptional machinery, Mediator complex probably can be considered as the second most important regulatory hub for different signaling networks in response to different developmental as well as environmental changes both in animals and plants as suggested by the work of many laboratories including ours. Before we describe the functions of individual Mediator subunit genes, the following is an account of changes in the transcript level of Mediator subunit genes in different tissues, and also how they are affected by different stages of growth and development. We have also discussed the changes in the transcript level of Mediator subunit genes in response to different hormones and abiotic stress treatments.

#### Tissue-specific and developmental regulation of mediator subunit genes

In an attempt to answer the question of what affect the levels of expression of individual Mediator subunits, analyses of differential expression of *MED* genes in different tissues and during different stages of plant development were performed. Several *MED* genes were significantly regulated during panicle and seed development stages as compared to root and leaf of the rice plants (Mathur et al., 2011). The enrichment of seed storage-specific promoter elements in certain *MED* genes raises possibilities of important function of MED subunits during embryo development and seed maturation. The increased abundance of *OsMED8* and *OsMED11_1* at early panicle and seed stages implicates their probable roles in reproductive development of rice. Middle module subunit, *AtMED21_1* showed approximately two-fold upsurge in the advanced stages of seed development which supports the reported role of AtMED21 in embryo development and cotyledon expansion. OsMED21 might be involved prominently in the early stages of panicle development (Dhawan et al., 2009; Mathur et al., 2011). OsMED31_1 is expressed more in leaf as compared to root. The tail module subunit, *AtMED14* is significantly expressed in leaf as compared to other parts of the plants, and has been implicated in the control of cell cycle duration and root elongation (Autran et al., 2002; Krichevsky et al., 2009). In rice, *OsMED26* is expressed more in root as compared to leaves. Significant up-regulation of *OsMED15_1* during different stages of seed development in different rice cultivars supports its probable role in seed development (Thakur et al., 2013). In this process, interaction of OsMED15_1 with seed-specific transcription factors could be predicted. SNP analysis of this gene sequence among several rice cultivars significantly segregated long and short grain varieties. Thus, an important gene regulatory role of this Mediator subunit in seed development and size determination is highly anticipated (Thakur et al., 2013). Several plant-specific Mediator subunits like *MED34, MED35, MED36*, and *MED37*, which have not been assigned to any module yet, are expressed more in reproductive stages as compared to vegetative parts implying its tissue-specific functions. The Mediator subunit, *MED36* is expressed more in the root of Arabidopsis and is anticipated to be involved in root-specific gene regulatory functions (Pasrija and Thakur, 2013). Thus, Mediator complex as a whole is a dynamic entity, and its composition may fluctuate in different tissues at different stages of growth and development.

#### Stress and hormone-induced regulation of mediator subunit genes

In the process of delineating how hormones affect the expression of different *MED* subunits in Arabidopsis, it was found that brassinosteroid (BR) and abscissic acid (ABA) have more significant impact on the transcription of *MED* genes as compared to other hormones including auxin and jasmonic acid (JA) (Pasrija and Thakur, 2012). *AtMED37*, which was discovered as a plant-specific Mediator subunit, is the most highly up-regulated *MED* in response to BR treatment. The reported 2.5-fold increase of *AtMED12* in response to BR treatment might also shed some light on its role in embryo development (Gillmor et al., 2010; Pasrija and Thakur, 2012). Among the other significant expression changes of Mediator subunits in response to phytohormone treatments, more than two-fold build-up in the transcript level of *AtMED18* in response to JA deserves special mention. AtMED18 has been reported to be involved not only in flower development but also in disease signaling (Zheng et al., 2013; Lai et al., 2014). On the other hand, we noted significant down-regulation (>40%) of tail module subunit genes, like *AtMED15, AtMED14*, and *AtMED5* in response to auxin treatment (Pasrija and Thakur, 2012). Although auxin and BR are known for their synergistic effects on plant growth and development, transcription of a set of Mediator genes was different in response to these hormones. For instance, *AtMED15* was up-regulated by BR but severely down-regulated by auxin. It seems that these two hormones show their transcriptional effects by a combination of different set of Mediator subunits (Pasrija and Thakur, 2012). In rice, there was not much effect on the transcript abundance of *MED* genes in response to different stresses like drought, salt, and salinity, but one, *OsMed37_6*, exhibits around two-fold change in response to different stresses (Mathur et al., 2011). However, in Arabidopsis, significant transcriptomic reprogramming of the Mediator subunit genes in response to high light, dark, and high salinity conditions was documented (Pasrija and Thakur, 2012). Interestingly, MED16 has been reported to be involved in cold signaling pathways, but the expression level of both *AtMED16* and *OsMED16* remains unchanged in response to cold treatment (Warren et al., 1996; Knight et al., 1999; Mathur et al., 2011). Like its role in cold signaling, more than two-fold increase of *MED16* transcript in response to salinity stress may imply its role as a converging point of both salt and cold signaling pathways. The important functions of AtMED12 in light and salt signaling pathways can not be ruled out because of its two-fold up-regulation in response to high light and salt conditions. Induction of *AtMED37* in response to BR and low light suggests a probable link between shade and BR signaling, and the process may be mediated by Endoplasmic Reticulum-Associated Degradation (ERAD) (Hong et al., 2008; Pasrija and Thakur, 2012). The up-regulation of *AtMED37* in response to cold and salinity stresses provokes an intriguing hypothesis that AtMED37 may act as an integrative hub of many different signaling pathways, which is supported by the near ubiquitous, high expression level of *AtMED37* in all the tissues tested so far (Pasrija and Thakur, 2012, 2013).

#### Compositional dynamics of mediator complex

Accumulating evidences suggest that Mediator complex is a dynamic and highly flexible entity, and its structural composition alters depending upon the context. Based on the spatio-temporal regulations of transcription of individual Mediator subunit genes in response to different stimuli, we predicted enrichment of specific structural arrangement composed of specific Mediator subunits during certain developmental stages (Pasrija and Thakur, 2013). However, as the Mediator stimulates basal transcription by participating in the recruitment of RNAP II at specific sites all over the genome, a basic, core structure should always be maintained irrespective of tissue, cell, development stage, or any environmental condition. That is the reason that transcription of a set of *MED* genes is not affected by hormones, stresses, or developmental cues. In animal cells, this has been well-illustrated by the presence of a simpler Mediator complex made of just 6–8 members in differentiated cells as compared to a 26-member Mediator complex in the cancerous and stem cells (Deato et al., 2008).

### Genetic and mutational analyses of mediator subunit genes

A large portion of total protein coding genes in eukaryotes requires the presence of Mediator complex even to sustain basal level of transcription. This proves unequivocally that Mediator constitutes important part of the basal transcriptional machinery. However, drastic morphological changes in mutants of individual Mediator subunits suggest that Mediator could also act as selective gene regulator both in metazoans and plants (Malik and Roeder, 2010; Taatjes, 2010; Kidd et al., 2011; Mathur et al., 2011; An and Mou, 2013; Poss et al., 2013; Allen and Taatjes, 2015). As the present review is plant specific, the following is an account and critical analyses of important functions of Mediator subunits reported from different plant species through mutational and genome-wide transcriptom analyses (Tables 1–**3**).

#### Embryonic development

In Arabidopsis and other plants, different phases of embryo development and maturation are marked by specific patterns and shapes. The Mediator subunits, MED12 and MED13, also known as GRAND CENTRAL (GCT) and CENTER CITY (CCT), respectively, mediate the embryo pattern formation, albeit in a transient manner (Gillmor et al., 2010). Mutations in these two genes disrupt the central and peripheral identity of the embryo along with the inhibition of globular to heart transition (Gillmor et al., 2010). Further investigations led to the prediction that the aberrant pattern during early embryo development might be due to a transient transcriptional repression of important genes like those encoding KANADI 1 and KANADI 2 transcription factors. AtMED13, also known as Macchi Bou2 (MAB2), has also been reported to be involved in embryo patterning and cotyledon development (Ito et al., 2011). In this case, the mutant shows aberrations in auxin response. The inability of *Atmed13* embryo to perceive and respond to auxin signals might account for its defective cotyledon formation. Recently, these two kinase module subunits have been shown to be involved in three more developmental transitions, i.e., germination, vegetative phase change, and flowering (Gillmor et al., 2014). Interestingly, the delay in vegetative phase change occurs largely due to over-expression of miR156 and the delay in flowering is caused by the increased production of *FLC*. On the whole, AtMED12 and 13 act as a global regulator of temporal genes making the developmental transitions a tightly controlled phenomenon.

#### Flower development

One of the most well-characterized Mediator subunits in plants is AtMED25, which has been described earlier as PFT1. MED25/PFT1 was discovered as a positive regulator of shade avoidance in Arabidopsis (Cerdán and Chory, 2003). It was postulated to be involved in control of flowering by phytochrome B pathway, which is dependent on light quality. The *Atmed25* plants flower late as compared to the wild type (Kidd et al., 2009). It has been demonstrated that AtMED25 positively regulates CONSTANT (CO) and FLOWERING LOCUS T (FT), two important flowering regulators in Arabidopsis (Inigo et al., 2012a). AtMED25 seems to be subjected to the phenomena of “activation by destruction.” AtMED25 is an unstable protein that is targeted by two RING H2 proteins, MBR1, and MBR2 for degradation by proteosomal pathway (Inigo et al., 2012b). The high turnover of AtMED25 is required for the activation of FT which promotes flowering. The phenomena elegantly demonstrate how a Mediator subunit follows “activation by destruction” principle to control a plant-specific event, and also adds a new dimension to Mediator function.

Few other Mediator subunits have also been implicated to be involved in flowering process (Table 1). Along with AtMED25, the delayed flowering phenotype was also observed in *Atmed8* mutants both under short and long day conditions (Kidd et al., 2009). In *Atmed8* mutants, the level of *FT*, a positive regulator of flowering, is low whereas *FLC*, a negative regulator of flowering, is expressed more. Enhanced phenotype in the double mutant of *Atmed25/Atmed8* suggests that AtMED25 and AtMED8 work independently and they might be controlling flowering process by responding to two different signaling pathways in synergy.

Mediator subunit MED12 (also known as CRYPTIC PRECOCIOUS, CRP) is a positive regulator of flowering and affects multiple genes working upstream and downstream of FT (Imura et al., 2012). As AtMED12 is a part of the kinase module and could interact with histone H3K9 methyltransferase, there is a possibility that it is involved in the epigenetic regulation of *FLC* and *FT* genes (Ding et al., 2008).

Very recently, another Mediator subunit, AtMED18, has been reported to be involved in flowering (Zheng et al., 2013; Lai et al., 2014). The loss-of-function mutant showed delayed flowering, and has altered level of *FLC* and *FT*. AtMED18 has been reported to interact with SUPPRESSOR OF FRIGIDA 4 (SUF4), and together binds to the promoter of *FLC* gene (Lai et al., 2014). Normally, AtSUF4 is a positive regulator of *FLC* gene. AtMED18 probably acts as suppressor of AtSUF4 activity.

The process of flowering requires the transition of vegetative primordia to reproductive primordia, and the region is marked with constant cell division. As the Mediator complex is often connected with dynamic cellular activities, it is quite obvious that Mediator plays significant role in the process of flowering and that is why several subunits affect this process (Table 1). Mostly, the loss-of-function mutations of Mediator subunits led to late and abnormal floral development, which is attributed to the perturbation in the transcript level of important flowering regulators like *FLC, FT*, and floral identity regulators like *AG*. But the missing link is how Mediator subunits control the expression of these genes. The non-coding RNAs play important role in epigenetic regulation of *FLC* gene (Crevillén and Dean, 2011; De Lucia and Dean, 2011). Given the reported association of non-coding RNA with the Mediator complex, a similar kind of mechanism in flowering time control could be envisaged (Lai et al., 2013).

#### Root development

A search for the role of Mediator subunits in root morphogenesis revealed the pivotal role of MED25 and MED8 in the production of root hairs in Arabidopsis (Sundaravelpandian et al., 2013; Raya-González et al., 2014). The absence of root hairs in *Atmed25* and *Atmed8* is due the inappropriate distribution of hydrogen peroxides (H_2_O_2_) and superoxides (${\text{O}}_{2}^{-}$) over the surface of tap root system. In fact, the comparison of the transcriptome of wild type and the *Atmed25* plants revealed that class III peroxidases are the worst affected ones in the mutant, perturbing the ROS homeostasis across the root length. The more severe phenotype of *med25/med8* double mutant eliminates the possibility of these two genes interacting in the same pathway. It will be interesting to find out if other MED subunits assist MED25 and MED8 in root hair development. Also, knowledge of transcription factors targeting these subunits will be helpful in understanding the mechanisms of transcriptional regulation of this process.

#### Other growth and developmental events

Mediator subunit CDK8 (or HEN3) of the kinase module plays important role in specification of stamen and carpel in Arabidopsis (Wang and Chen, 2004). Mechanistically, like in yeast and mammals, AtCDK8 phosphorylates the CTD domain of largest subunit of RNAP II and represses transcription. This leads to an enhanced expression of *AG, AP1*, and *AP2* in cdk8 mutant. CDK8 is abundantly expressed in the proliferating tissues suggesting its involvement in mediating cell division and cell fate specification. Alternatively, as the RNA transcription and RNA processing are coupled and CDK8 interacts with CTD domain of RNAP II, the perturbed alternative transcript of *AG1* in the mutant plant indicates its probable role in alternative splicing. What it warrants at this moment is to identify the transcription factors that interact with these Mediator subunits and the immediate target genes for a better understanding of the regulatory circuitry that controls cell number and size.

Another Mediator subunit AtMED18 contributes to the organ identity and number. Other than being short in stature and late flowering, *Atmed18* plants have altered number of floral parts. In mutant plants, sepals and petals are more and anthers are less. There are two carpels, and the pollen maturation is delayed (Kim et al., 2011; Zheng et al., 2013). The down-regulation of floral homeotic genes like *AP1, PI*, and *AG* in *Atmed18* mutant plants indicates crucial regulatory role of AtMED18 in homeotic gene expression (Zheng et al., 2013). Additionally, AtMED18 may control the organ identity genes through its association with HEN3/CDK8, which also controls organ identity and shows similar loss-of-function phenotypes (Wang and Chen, 2004).

Cell number over the entire arial parts of Arabidopsis is decreased if there is a mutation in another Mediator subunit *MED14*, more popularly known as *STRUWWELPETER (SWP)* (Autran et al., 2002). Both the leaf number and size in the heterozygous mutant lines are reduced whereas homozygous mutant lines are sterile. The importance of *AtMED14* in leaf development is also evident by its strong expression in leaves. The mutant plant also carries a disorganized Shoot Apical Meristem (SAM). The arrest of cell division in *Atmed14* plants may result from the endoreduplication of the chromosomal DNA. Mechanistically, AtMED14 may interact with SMP1, SMP2 which encode step II splicing factors as both the mutants show similar phenotypes (Clay and Nelson, 2005). LEUNIG, a GroTLE transcription corepressor, has been reported to interact with AtMED14 and controls multiple physiological processes (Gonzalez et al., 2007).

MED4 is a subunit in the middle module, and has recently been speculated to be involved in growth of the tillers in rice (Li et al., 2014). Its homozygous mutants are embryonic lethal. Surprisingly enough, it interacts with SAD1, an ortholog of RNA polymerase I subunit RPA 34.5 in rice, and is involved in rRNA biosynthesis. It is worth mentioning here that SAD1 was isolated as a component of Mediator complex during the complex purification study in Arabidopsis (Bäckström et al., 2007). It also interacts with the counter parts of the other RNA polymerases like pol II and pol III. Thus, this is the first example which shows the interaction between the Mediator complex and the RNA pol I and III, and thus extends the function of Mediator beyond RNAP II-mediated transcription.

Cell proliferation and cell expansion are two important basic processes in any organism, which ultimately determine the organ size, hence the entire body size. DA1 is an ubiquitin receptor and restricts cell proliferation to control final size of organs in Arabidopsis (Li et al., 2008). In a genetic screen to find the enhancer of *DA1* mutation, *AtMed25* mutant was characterized (Xu and Li, 2011). AtMED25 too negatively controls the cell proliferation and cell enlargement. Loss-of-function mutant of *MED25* has large organs, with larger and slightly increased numbers of cells as a result of an increased period of cell proliferation and cell expansion. The observed phenotype in *Atmed25* mutant plants may be partly because of the up-regulation of expansin genes like *AtEXP1, AtEXP3, AtEXP5, AtEXP9, AtEXP11*, and *AtEXPB3*. Consistent to this, plants over-expressing *MED25* have small organs owing to decrease in both cell number and size. Further analysis eliminated the possibility of higher ploidy level in the mutant plants as the cause of larger organ size. The genetic and physiological data suggest that MED25 acts to limit cell and organ growth independently of its involvement in phytochrome and JA signaling pathways (Cerdán and Chory, 2003; Kidd et al., 2009; Xu and Li, 2011; Chen et al., 2012; Inigo et al., 2012a,b). Rather, MED25 functions synergistically with DA1 to control organ growth by restricting cell proliferation. In contrast to MED25, MED8 positively controls the organ size (Xu and Li, 2012). The mutant *Atmed8* plants have shorter flowers because of reduced cell expansion. Analysis of *med25med8* double mutants revealed the antagonistic behavior of MED25 and MED8, at least in the case of cell expansion and cell proliferation, hence in organ size determination.

Getting rid of lignins from the crops for its usage as forage, pulp, and paper production poses a significant challenge because most of the lignin related mutants are stunted and growth defective. Two such mutants, *ref8-1* and *ref8-2*, which are deficient in lignin content, are short and display little vegetative growth. Two Mediator subunits, AtMED5a (REF4), and AtMED5b (RFR1), have been shown to negatively regulate plant height and lignin content (Bonawitz et al., 2012). Interestingly, the mutants of either of these subunits rescue the phenotype of *ref8-1* or *ref8-2* without any yield penalty on biomass production (Bonawitz et al., 2014). Importantly, the mutants are free from biomass recalcitrance. Thus, the domain of Mediator function also encompasses the regulation of cell wall biosynthesis, which is of great practical value.

Iron is one of the essential elements in plants, and its uptake and assimilation are tightly controlled. Two Mediator subunits, AtMED16 (YID), and AtMED25, have been reported to control iron homeostasis is plants (Yang et al., 2014; Zhang et al., 2014). The mutants of these Mediator subunits display hypersensitivity toward iron deficiency resulting in leaf chlorosis. AtMED16 directly interacts with FIT, the master regulator of iron homeostasis in plants. In chromatin immunoprecipitation analysis, AtMED16 was found to be present on the promoter of the iron acquisitions genes like *FRO2* and *IRT1*, probably by interacting with FIT (Zhang et al., 2014). FIT also interacts with other bHLH proteins forming heterodimers and these heterodimers bind to *FRO2* and *IRT1* promoters. The binding of AtMED16 probably confers stability to the FIT/bHLH complex (Zhang et al., 2014). On the other hand, MED25 interacts with two transcription factors, EIN3 and EIL1, which are involved in ethylene signaling. EIN3 and EIL1 directly interact with FIT. FIT is a highly unstable protein and the interaction of MED25 with EIN3 and EIL1 provides stability to FIT enabling it to regulate downstream iron regulatory genes like *FRO2* and *IRT1* (Yang et al., 2014). Interaction between AtMED16 and AtMED25 has also been reported (Zhang et al., 2014). However, the effects of double mutations of these two genes are yet to be investigated. Probably, AtMED16, AtMED25, EIN3, EIL1, FIT, and other bHLH proteins form a stable activator complex on the promoter of *FRO2* and *IRT1* leading to their activation during iron deficient conditions.

#### Defense signaling

Plants in its natural environments are being constantly challenged by myriad of insect pests and pathogens, which together constitute the biotic stresses. A survivor plant activates its defense arsenal quickly and efficiently in order to counter the invading and inflicting biotic agents. Such an orchestrated and rapid response is only achievable by the timely activation of key defense genes. Emerging reports have established Mediator complex as an essential component for regulation of genes involved in defense pathways (An and Mou, 2013). In comparison to other pathways, higher number of Mediator subunits has been shown to be involved in defense signaling (Table 2).

**Table 2:** **Plant Mediator subunits involved in stress signaling**.

**Biotic and abiotic stresses**	**Gene name**	**Functions in brief**	**Interacting proteins**	**References**
Biotic stress	*AtMED25/PFT1*	Regulates the jasmonate pathway	MYC2, AP2/ERF, bHLH, MYB, WRKY, bZIP	Kidd et al., 2009; Çevik et al., 2012; Chen et al., 2012
	*AtMED8*	Same as AtMED25. The mutant shows more disease susceptibility as compared to AtMED25	Unknown	Kidd et al., 2009
	*AtMED16/SFR16*	Involved in SA and JA pathway of disease signaling	Unknown	Wathugala et al., 2012; Zhang et al., 2012
	*AtMED21*	Provides resistance against the necrotrophic fungal pathogens	HUB1	Dhawan et al., 2009
	*AtMED15/NRB4*	May be involved in SA response pathway of disease signaling. No specific transcriptomic changes observed in mutant plants	Unknown	Canet et al., 2012
	*AtMED14/SWP*	Effectors of SAR were down-regulated in mutant plant. Both positive and negative regulators of SAR pathway were affected significantly in *Atmed14* mutant	Unknown	Zhang et al., 2013b
	*AtMED19a*	Provides resistance against powdery mildew pathogen, *Hyaloperonospora arabidopsidis*	HaRxL44	Caillaud et al., 2013
	*AtCDK8*	Binds with the promoter of the *AGMATINE COUMAROYLTRANSFERASE* to increase expression of defense active bio-compounds	AtMED25	Zhu et al., 2014
	*AtMED18*	Plays positive regulatory role in defense signaling inhibiting the expression of disease susceptibility genes, glutaredoxins and thioredoxin	YIN YANG1 (YY1)	Lai et al., 2014
Abiotic stress	*AtMED25*	AtMED25 controls salinity stress and drought stress antagonistically	DREB2A, ZFHD1, and MYB	Elfving et al., 2011
	*AtMED16/SFR16*	The mutant plants are defective in cold acclimation	Unknown	Knight et al., 1999, 2008, 2009; Hemsley et al., 2014

The first Mediator subunit reported to be involved in defense response was AtMED25 (Kidd et al., 2009). AtMED25 bears similarity with the mammalian MED25, which also plays important role in defense response (Leal et al., 2009). In Arabidopsis, MED25 directly affects JA-dependent gene expression (*PDF1.2, HEL, CHIB*, and *ESP*), and provides resistance against the leaf-infecting necrotrophic fungi, *Alternaria brassicicola*, and *Botrytis cinerea* (Kidd et al., 2009). The complementation of *Atmed25* by its homologs from wheat strengthened the view that functions of some of the Mediator subunits may be conserved in higher plants (Kidd et al., 2009). A group of 12 transcription factors (TFs) have been shown to interact with AtMED25, which includes AP2/ERF, bHLH, MYB, WRKY, and bZIP. Among these transcription factors, many have previously been demonstrated to be involved in JA signaling pathway (Çevik et al., 2012). Furthermore, AtMED25 takes part in ERF1- and ORA59-dependent activation of *PDF1.2* gene as well as MYC2-dependent activation of *VSP1* gene, which are some important genes in the JA signaling pathway (Çevik et al., 2012). In fact, MED25 physically associates with the bHLH transcription factor, MYC2 in promoter regions of its target genes to elicit a positive effect on their transcription (Chen et al., 2012). The head module subunit mutant, *Atmed8*, behaves like *Atmed25* but shows pronounced susceptibility toward *A. brassicicola* (Kidd et al., 2009). These two mutants, however, do not interact genetically, suggesting that AtMED25 and AtMED8 might be acting in two independent pathways controlling the same response and phenotype (Kidd et al., 2009).

The middle module subunit MED21 is an essential requirement for survival of Arabidopsis plants as its T-DNA insertional homozygous lines are embryonic lethal (Dhawan et al., 2009). The RNAi lines of *MED21* are highly susceptible to *A. brassicicola* and *B. cinerea.* The detailed study revealed that MED21 interacts with RING E3 ligase, Histone Monoubiquitination1 (HUB1), which mediates the H2B ubiquitination, thus establishing a link between Mediator and the chromatin remodeling. The induced expression of both *MED21* and *HUB1* in response to chitin treatment, an important constituent of fungal cell wall, suggests their probable role in defense signaling (Dhawan et al., 2009).

The head module subunit, AtMed19a interacts with nuclear localized fungal effector (HaRxL44) of powdery mildew pathogen, *Hyaloperonospora arabidopsidis* (Hpa). This leads to proteasome-dependent degradation of AtMed19a and shift the balance from SA-mediated disease resistance to ET/JA-mediated transcriptomic changes making the plants more vulnerable to bitrophs (Caillaud et al., 2013). This highlights how pathogens can break plant immune barrier by hijacking the important resistance mechanisms offered by Mediator complex. Another head module subunit, AtMED18, plays a positive regulatory role toward necrotropic fungal infection by interacting with YYI keeping the expression of glutaredoxin and thioredoxin genes suppressed (Lai et al., 2014).

Three tail module subunits, AtMED14, AtMED15, and AtMED16 have been reported to be involved in defense signaling as well (Canet et al., 2012; Wathugala et al., 2012; Zhang et al., 2012, 2013b) (Table 2). The Arabidopsis plants carrying mutation in *MED16* are compromised for SA- and JA-dependent defense responses (Wathugala et al., 2012). The *Atmed16* mutant plants are more susceptible to *Pseudomomas syringae* attack, and exhibit lower expression of defense-related genes like those coding for PR (Pathogenesis Related) proteins and defensins. Moreover, the expression levels of the important SAR (systemic acquired resistance) markers like *PR1, PR2, PR5, GST11, EDR11, SAG21* are severely reduced in *Atmed16* mutant (Zhang et al., 2012). Hence, MED16 acts as a positive regulator of SA-induced gene expression. Similarly, the *Atmed16* mutation also blocks the induction of the JA/ET-dependent gene expression making the plants vulnerable to necrotrophic fungi like *A. brassicicola* and *B. cinerea* (Zhang et al., 2012). Thus, MED16 seems to function as an integrative hub for both SA and JA signaling pathways. The tail module subunit, AtMED15, also dubbed as NRB4 (Non-recognition of BTH4, a salicylic acid analog), has recently been shown to be involved in defense signaling via its involvement in SA pathway (Canet et al., 2012). The mutant plants with defective MED15 do not show any noticeable phenotypic change except its attenuated response to SA, reminiscent of the effects of *npr1* mutation in plants' defense signaling. NPR1 (non-expresser of *PR* genes) plays a pivotal role and takes the center stage in the SA-mediated defense pathways (Dong, 2004). However, neither a genetic nor a biochemical interaction has been reported between MED15 and NPR1. The additive phenotypes of *Atmed15/npr1-70* plants indicate that they might work at different point of SA signaling pathway. Moreover, *Atmed15* affects neither the localization of NPR1 nor its stability. Thus, mechanistically, MED15/NRB4 might be functioning downstream of NPR1 in the regulation of SA response pathway. The exact position of MED15 in SA signaling pathway is not known, and it warrants detailed molecular and genetic investigations. A mutation in *AtMED14* subunit gene suppresses the SA-dependent expression of defense genes (Zhang et al., 2013b). AtMED14 prevents *PR1* expression without interfering the binding of NPR1, the master regulator of defense gene expression, to its promoter. This leads to the speculation that AtMED14 might be responsible for the recruitment of RNAP II to the promoter of *PR1* gene. Further investigation is needed to delineate the exact mechanism involved in the process. Thus, it seems that most of the subunits in the tail module play significant role in the regulation of defense gene expression during pathogen attack. However, the mechanisms employed by the three different Mediator subunits (MED14, MED15, and MED16) differ considerably toward controlling the expression of defense genes. The *Atmed16* mutation differentially affects the expression of different positive and negative regulators of SAR, whereas *Atmed14* mutation inhibits expression of similar genes. Moreover, defense-related transcriptomic change in the case of *Atmed14* is much smaller as compared to that in the case of *Atmed16*.

The kinase module component, AtCDK8, has recently been reported to be a positive regulator of disease response (Zhu et al., 2014). The mutant plants are highly susceptible to *A. brassicicola*. Mechanistically, it interacts with another Mediator subunit, AtMED25, and regulates JA-mediated gene expression during pathogen signaling. Additionally, it binds with the promoter of *AGMATINE COUMAROYLTRANSFERASE (AACT1)* gene whose products are involved in the biosynthesis of defense active bio-compounds like hydroxycinnamic acid amides in plants.

#### Abiotic stress signaling

Plants are sessile organisms. They cannot run away to safer places during inclement weather. On the other hand, growth and development of the plant is profoundly influenced by the environment. A robust, surviving plant must translate the vagaries of the surrounding environments into proper signals relaying them to the transcriptional machinery ensuring the adaptability of the plants to the changed milieu. Of late, Mediator has emerged as an integrative hub for the different signaling pathways leading to the transcription regulation by RNAP II. So it is highly anticipated that the Mediator will also play a crucial role in the integration of signals originated in response to stresses like drought, cold, salinity etc. So far two Mediator subunits (Table 2), which also play important roles in biotic stresses, have been reported to be involved in abiotic stress signaling. The *Atmed25* mutant seeds display increased sensitivity toward salt stress during germination. The importance of MED25 in high salinity is conserved across the plant species (Elfving et al., 2011). In a yeast two hybrid screen, three stress-specific transcription factors, DREB2A, ZFHD1, and MYB like proteins were found to be interacting with the ACID (Activator Interacting Domain) domain of AtMED25. The plants carrying mutations in any of these genes also display severe salt sensitivity. Mechanistically, ACID domain of MED25 might be targeted by these transcription factors for communication with the RNAP II transcriptional machinery for effective salt-responsive transcriptomic changes in plants. Surprisingly, MED25 negatively regulates drought tolerance in plants (Elfving et al., 2011). The mutant plants display huge increase in the expression level of drought responsive marker genes like *RD29A, RD29B*, and *DREB2A*. AtMED25 has been projected as a co-repressor interacting with the repressor domain of DREB2A making the plants vulnerable to drought stress (Elfving et al., 2011). Thus, it is one of those examples, where the same Mediator subunit, AtMED25, controls salt and dehydration stresses in an antagonistic manner.

MED16, originally discovered as SFR6 in Arabidopsis before being identified as a part of Mediator complex, has been reported as an important component involved in acclimation to cold (Knight et al., 1999, 2008; Wathugala et al., 2011). The mutant plants fail to embrace freezing temperature following its exposure to subzero temperature. At the molecular level, the plants are incapable of switching on the COR (cold on regulation) regulon including the expression of *LTI78, COR15A*, and *KIN1/2*. Microarray analysis revealed that a subset of cold-responsive genes bearing CRT/DRE motifs in their promoter regions gets miss-regulated in *Atmed16* mutant plants (Knight et al., 1999). These genes are involved in freezing tolerance and controlled by CBF transcription factors (Boyce et al., 2003). However, neither the expression of CBF nor its localization is affected in *Atmed16* mutant plants (Knight et al., 2009). Thus, it provokes the intriguing speculation that MED16 might modulate the activity of CBFs through post-transcriptional modulation.

#### Associated nuclear functions

One of the most significant discoveries of Mediator function in plants is related to miRNA and siRNA biogenesis (Kim et al., 2011). The loss-of-function mutants of three Mediator subunits, *Atmed17, Atmed18*, and *Atmed20a*, are short in stature, late flowering, and bear small fruits as compared to the wild types. The in-depth, detailed analyses revealed that these mutants are defective in the regulation of miRNA and siRNA at the transcriptional level. The occupancy of RNAP II at the promoters of miRNA and siRNA genes was also highly reduced in these mutants. The role of these Mediator subunits has also been implicated in the silencing of transposons and repeat sequences. These elements normally undergo siRNA-mediated transcriptional gene silencing, and were de-repressed in *med17, med18*, and *med20a*. On the other hand, co-purification of MED36 with the largest subunit of RNA pol V led to the intriguing hypothesis that Mediator complex may act in cooperation with other RNA polymerases in the production of non-coding RNA (Huang et al., 2009). Although it is a matter of debate, the same study also advocated the role of the Mediator complex as a general transcription factor. The discovery brought a paradigm shift in the understanding of Mediator functions beyond the regulation of subunit specific functions (Table 3).

**Table 3:** **Plant Mediator subunits involved in associated nuclear functions**.

**Associated nuclear functions**	**Gene name**	**Functions in brief**	**Interacting proteins**	**References**
	*AtMED17, AtMED18*, and *AtMED19*	Regulation of miRNA and siRNA biogenesis by preventing the binding of RNAP II on the promoters of these genes	Unknown	Kim et al., 2011
	*AtMED34/AtRecQ2*	Involved in replication related phenomena like D-loop and Holiday structure disruption, maintenance of genomic stability	Unknown	Kobbe et al., 2008
	*MED36/FIB2*	Processing of rRNA by regulating its methylation	AtPRMT1a and AtPRMT1b (Probable)	Barneche et al., 2000; Yan et al., 2007; Huang et al., 2009
	*MED37a/BiP*	Helps in female gametophyte development mediating polar nuclei proliferation. Promotes degradation of BRI1-5	BRI 1-5 (Brassinosteroid Receptor)	Hong et al., 2008; Maruyama et al., 2010
	*AtMED 35/AtPRPa*	Probably takes part in RNA processing	CTD domain of RNAP II	Kang et al., 2009

The newest entrants into the expanding list of plant Mediator subunits are MED34 to MED37 (Bäckström et al., 2007). The phenomenon that provokes curiosity is that a DNA helicase, AtRecQ2, which takes part in replication related phenomena like genome stability, D-loop and Holliday structure disruption, turned out to be MED34 (Kobbe et al., 2008). The Arabidopsis Mediator subunit, MED36/FIB2 has been shown to encode a Fibrillarin (FIB2), which is involved in rRNA processing (Barneche et al., 2000). It interacts with and is methylated by histone methyltransferases, AtPRMT1a and AtPRMT1b, and co-purified with RNA pol V (Yan et al., 2007; Huang et al., 2009). MED37a (also known as BiP) was first characterized as one of the HSP70 family members, and is homologous to yeast Ig-binding protein (Rose et al., 1989). It is involved in polar nuclei fusion during female gametophyte development, and is essential for the regulation of endosperm nuclei proliferation (Maruyama et al., 2010). In Arabidopsis, it also interacts with BR hormone receptor, BRI1, facilitating its proteasome-independent endoplasmic reticulum–associated degradation (ERAD) (Hong et al., 2008). Among the three AtPRP40s (*Arabidopsis thaliana* pre-mRNA processing protein 40), AtPRP40a has been recently named as AtMED35 of the Mediator complex. It interacts both with the phosphorylated and the unphosphorylated forms of the largest subunit of RNAP II. In Arabidopsis, it has its characteristic high expression level in roots and cauline leaves as compared to the other parts. The mutant does not show any phenotype, probably because of its redundancy with AtPRP40b and AtPRP40c (Kang et al., 2009).

The key importance of the Mediator complex lies in its ability to act as an adaptor molecule between transcription factors and the RNAP II, and hence the on-going research has so far been directed toward its role in the initial processes of transcription. The recent findings regarding its probable role in elongation and termination have not only expanded its arena of functionality, but have given fresh impetus toward the possibility of involvement of Mediator complex in other co-transcriptional processes like RNA processing (splicing, capping, polyadenylation), alternative splicing and epigenetic regulation (Table 3). Hence, the functional association of some Mediator subunits in these processes seems quite natural, and these issues need to be addressed more critically in future. As expected Mediator complex has critical control over miRNA and siRNA biogenesis as these are also transcribed by RNAP II. However, the association of other RNA polymerases with the Mediator complex, and its role in other RNA polymerase-mediated transcriptional events need to be examined further. Currently, we lack explanations for the Mediator subunits, which take part in phenomena like replication, protein degradation etc.

## Complex system of mediator as target of diverse transcription factors to regulate different processes and pathways

Mediator acts as an intermediary between the *cis*-element bound transcription factor and the RNAP II-mediated transcriptional machinery relaying the information from the transcription factor to the transcription apparatus. Recently, a couple of reports in plants have made the picture more complicated as the interaction between the transcription factor and the Mediator complex is not a simple binary one-one interaction. The Arabidopsis Mediator subunit, MED25 can interact with several transcription factors (DREB2A, ZFHD1, and MYB like proteins), that function in the same pathway. The mutants of all these three genes show increased sensitivity to salinity stress (Elfving et al., 2011). On the other hand, it has been also shown that AtMED25 can differentially control two seemingly different pathways, JA and ABA signaling, by interacting with two different transcription factors like MYC2 and ABI5, respectively (Chen et al., 2012). Similarly, AtMED18 has been shown controlling multiple plant responses by interacting with different transcription factors (Lai et al., 2014). An analogous situation also happens in yeast where different nuclear receptor-like transcription factors like Oaf1, Pdr1, and Pdr3 target the same Mediator subunit, MED15, to control different processes like fatty acid metabolism and multi-drug resistance (Thakur et al., 2008, 2009). On the contrary, a single characteristic/phenotype in plants can also be controlled by the concerted actions of more than one Mediator subunits (Tables 1, 2). Detailed investigation is needed to figure out whether these Mediator subunits do take part in the same developmental pathway while controlling a specific character or they control different developmental programs converging to a single phenotype. There are copious examples in animals where distinct Mediator subunits can control specific developmental and signaling pathways (Ito et al., 2000; Stevens et al., 2002; Ge et al., 2008). We suggest that the permutations and combinations of transcription factors with the Mediator subunits probably generate a Mediator code which dictates the downstream gene expression phenomena in co-ordination to the developmental stage and the prevailing environmental conditions. It might also involve Mediator complex undergoing a great deal of structural adjustment and alignment after binding with the transcription factors, which need to be studied in detail in the future.

## Conclusion

The universality of the Mediator complex in the transcription of protein coding genes has ushered a new era in the understanding of transcriptional regulations in yeast and human. The plant science community is not lagging far behind in Mediator research. The achievement includes not only the first Mediator complex isolation from Arabidopsis but also the discovery of ubiquitous presence of Mediator complex in almost all the phyla of plant kingdom.

A general revelation from different studies is that the repertoire of Mediator subunits has been expanded in plant species to cope up with the increased number of plant transcription factors. This provides better resilience power to the sessile plants against the vagaries of the biotic and abiotic stresses. However, a note of caution should be shown regarding the discovery of new Mediator subunits. Until now there are no defined parameters to designate a protein as Mediator subunit. The Mediator acts as a scaffold for the interaction of a number of transcriptional regulatory proteins. Does mere co-purification with the Mediator complex qualify a protein to be regarded as Mediator subunit? Recently, six new plant-specific Mediator subunits (AtMED32–AtMED37) were discovered in Arabidopsis, but later AtMED32 and AtMED33 were found to be AtMED2 and AtMED5, respectively. As some of their functions are not directly related to Mediator functions or transcription (as for example, AtMED34 or AtRecQ2), concern has been expressed regarding how truly these proteins represent Mediator subunits.

Many of the Arabidopsis Mediator subunits were characterized earlier, but not in consideration of its Mediator membership. Over the time, several Mediator subunits have been characterized in Arabidopsis and many more may follow. In most of the cases, phenotypes of a particular Mediator subunit mutant has been described, but its association with transcription factors and the set of genes under its control are yet to be discovered in majority of the cases. What is lacking more is the understanding of how the Mediator subunits interact with components of the basic transcriptional machinery resulting in the regulated transcription.

Recently, many of the hitherto unknown but interesting functional aspects of Mediator has been unveiled in other organisms further broadening the horizon of its roles. Mediator not only takes part in the recruitment of RNAP II on the promoters of the active genes but also in transcription elongation and termination, chromatin remodeling, alternative splicing, small, and long non-coding RNA biogenesis, heterochromatin formation. All these developments are taking place in the arena of yeast and metazoan biology. Except characterization of few Mediator subunits, studies involving the Mediator complex as a whole or the mechanistic dexterity of Mediator complex in general or gene-specific regulation has not been addressed with proper emphasis and interest in plants. So, besides characterization of the every Mediator subunits in model species, attention should also be focused to address how the Mediator controls different steps of transcription in terms of mechanical intricacies.

Presence of more than one paralog has been reported for some Mediator subunits. Another level of complicacy may arise regarding which paralog remains with the complex, which most probably is controlled in a temporal and spatial manner. The presence of more than one paralog at a time in the Mediator complex has not been reported by any group. The more interesting question which has just been started to be answered is how stable is the Mediator structure in terms of its subunit composition. We postulate that the structure of Mediator complex changes depending on the composition of Mediator subunits, which again is controlled by different biotic and abiotic stimuli. Mediator complex isolation and its structural comparison from different stages of growth and development hold the key to the questions of how the structural shifts due to changes in Mediator composition are translated into transcriptomic changes of a species in response to intrinsic and extrinsic factors. Armed with the tools of modern molecular biology like TAP, MudPIT, LC-MS/MS, and HT-ChIP; the aforementioned questions are anticipated to be answered at an accelerated speed in near future.

### Conflict of interest statement

The authors declare that the research was conducted in the absence of any commercial or financial relationships that could be construed as a potential conflict of interest.

## Abbreviations

MED: Mediator
RNAP II: RNA Polymerase II
BR: Brassinosteroid
SA: Salicylic acid
JA: Jasmonic acid
ET: Ethylene
MudPIT: Multi-dimensional Protein Identification Technology
TAP: Tandem Affinity Purification
LC-MS/MS: Liquid Chromatography-Mass Spectrometry
HT-ChIP: High-Throughput Chromatin Immunoprecipitation.
